# GABAergic neurons of the medial septum play a nodal role in facilitation of nociception-induced affect

**DOI:** 10.1038/srep15419

**Published:** 2015-10-21

**Authors:** Seok Ting Ang, Andy Thiam Huat Lee, Fang Chee Foo, Lynn Ng, Chian-Ming Low, Sanjay Khanna

**Affiliations:** 1 Departments of Physiology, Yong Loo Lin School of Medicine, 10 Medical Dr, Singapore; 2 Departments of Pharmacology, Yong Loo Lin School of Medicine, 10 Medical Dr, Singapore; 3 Departments of Anaesthesia, Yong Loo Lin School of Medicine, 10 Medical Dr, Singapore; 4 Neurobiology Programme, Life Sciences Institute, National University of Singapore, 21 Lower Kent Ridge Rd, Singapore

## Abstract

The present study explored the functional details of the influence of medial septal region (MSDB) on spectrum of nociceptive behaviours by manipulating intraseptal GABAergic mechanisms. Results showed that formalin-induced acute nociception was not affected by intraseptal microinjection of bicuculline, a GABA_A_ receptor antagonist, or on selective lesion of septal GABAergic neurons. Indeed, the acute nociceptive responses were dissociated from the regulation of sensorimotor behaviour and generation of theta-rhythm by the GABAergic mechanisms in MSDB. The GABAergic lesion attenuated formalin-induced unconditioned cellular response in the anterior cingulate cortex (ACC) and blocked formalin-induced conditioned place avoidance (F-CPA), and as well as the contextual fear induced on conditioning with brief footshock. The effects of lesion on nociceptive-conditioned cellular responses were, however, variable. Interestingly, the lesion attenuated the conditioned representation of experimental context in dorsal hippocampus field CA1 in the F-CPA task. Collectively, the preceding suggests that the MSDB is a nodal centre wherein the GABAergic neurons mediate nociceptive affect-motivation by regulating cellular mechanisms in ACC that confer an aversive value to the noxious stimulus. Further, in conjunction with a modulatory influence on hippocampal contextual processing, MSDB may integrate affect with context as part of associative learning in the F-CPA task.

Pain is debilitating with a cognitive and affective burden. Such an unpleasant experience is mediated by a variety of cortical and sub-cortical sites, including the anterior cingulate cortex (ACC), hippocampus and the amygdala. In this regard, experimental lesion or pharmacological manipulation of the ACC and the basolateral amygdala (BLA) indicate that these regions selectively attenuate aversion, induced on conditioning with the algogen, formalin, without attenuating acute nociception while manipulation of the hippocampus modulates formalin-induced acute nociceptive behaviours^1^,^2^,^3^,^4^. Interestingly, each of the above area is interconnected with the basal forebrain, including the medial septum diagonal band region (MSDB), which is also implicated in affect^5^,^6^,^7^,^8^,^9^,^10^,^11^. Indeed, more recent evidence suggests that the region facilitates acute nociception in the formalin model of persistent inflammatory pain^9^. However, at present it is not known whether the region modulates aversion as well.

Furthermore, the MSDB region is home to neurochemically diverse populations of neurons which includes, among others, cholinergic and parvalbumin-positive GABAergic neurons that together comprise the majority of the efferent projection from the basal forebrain^12^,^13^,^14^. Functional details, such as the role of different neural population of MSDB in modulation of various facets of nociception are, however, not well understood at present. Indirect measures suggest that the MSDB GABAergic mechanisms are activated in formalin nociception. For example, formalin injection induces hippocampal theta wave activity, a 3–12 Hz extracellular sinusoidal waveform that reflects oscillatory discharge of neurons in processing of information^15^,^16^. The hippocampal theta wave activity is modulated partly by intraseptal GABAergic mechanisms and septal GABAergic efferent and is integral to the mnemonic, sensorimotor and affective functions of the septo-hippocampus^6^,^15^,^16^,^17^. Based on the foregoing, we hypothesised that the MSDB GABAergic mechanisms are crucial to the noxious-aversive stimuli-induced behaviours, at least in part.

To test the hypothesis, we have performed experiments in which we have examined the effect of perturbation of intraseptal GABAergic transmission and/or the destruction of MSDB GABAergic neurons on acute and conditioned behaviours induced with persistent and brief noxious-aversive stimuli, namely formalin injection and footshock, respectively. Alongside, we have performed the following experiments to explore the system and network level changes that accompany MSDB manipulations: (a) effect of intraseptal bicuculline on animal exploration and hippocampal theta activation, (b) effect of septal lesion on affect-related cellular responses including unconditioned response in ACC to formalin injection^18^ and conditioned response in dorsal hippocampus CA1 and BLA that are implicated in aversive conditioned behaviours such as contextual fear (ctxtFC)^19^,^20^,^21^,^22^,^23^.

## Results

### Effects of intraseptal microinjection of bicuculline on hippocampal field activity and animal behaviours

**Figure 1:**
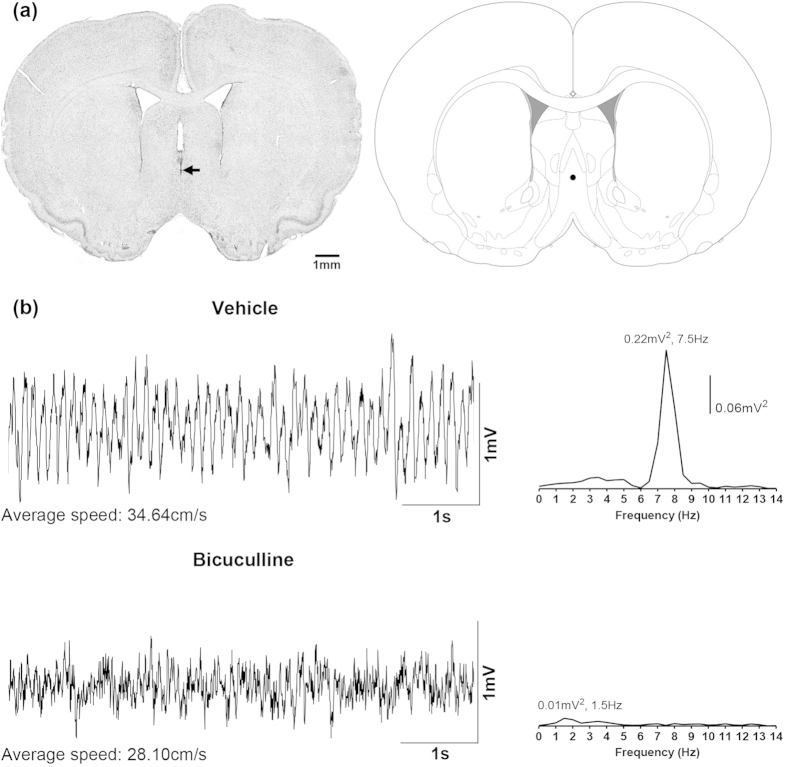
Microinjection of bicuculline into the medial septum attenuates hippocampal theta wave activity in the awake rat during exploration in open field. (**a**) Digital image of a Nissl-stained coronal section (left panel) and its corresponding diagrammatic representation (right panel). The coronal section is taken through medial septum and shows the microinjection site which is given by the dye spot (arrow). The diagrammatic representation is adapted from Paxinos and Watson^53^. The filled circle on the diagrammatic representation corresponds to the microinjection site on the digital image and was judged to be within the medial septum. (**b**) are the representative waveforms of hippocampal field activity recorded from stratum radiatum of the hippocampus. The waveforms were recorded during exploration in the first minute following placement of the animal into the open field chamber. The average speed of ambulation in 10-s period around the trace is given underneath each trace. Animals were microinjected with vehicle (top; 0.5 μl) or bicuculline (bottom; 0.125 μg/0.5 μl) into the medial septal region immediately prior to the start of the open field experiment. Clear theta wave activity was observed in the vehicle-injected animal and, in line with visual observation, the peak frequency of the associated Fast Fourier Transform (FFT; frequency resolution 0.5 Hz; on the right of each trace) of the hippocampal field activity was in the theta range (4–12 Hz) and was clearly demarcated from other frequencies around the region of the peak. However, microinjection of bicuculline resulted in a loss of theta wave activity and FFT analyses revealed a peak in the non-theta range.

**Figure 2:**
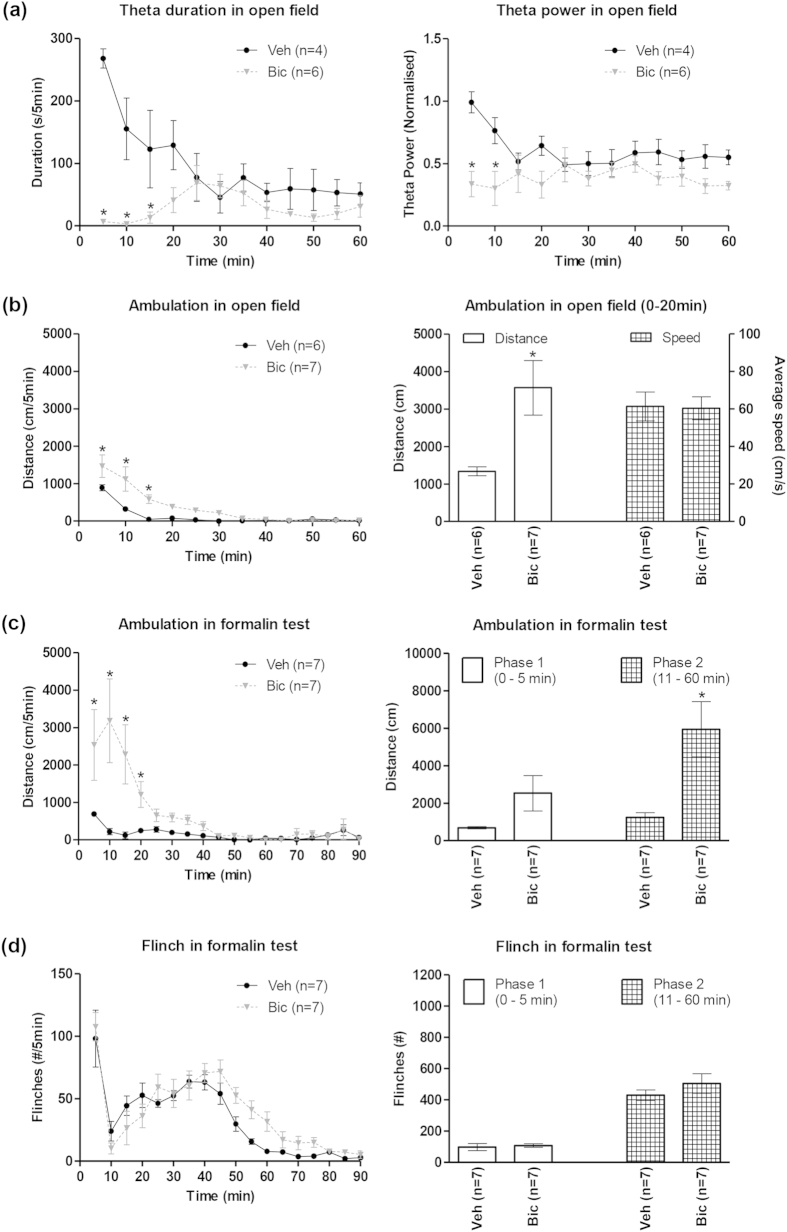
Intraseptal bicuculline affects hippocampal theta and ambulation, but not nociceptive behaviours. Intraseptal bicuculline (Bic; 0.125 μg/0.5 μl) attenuated duration of theta and theta peak power evoked during exploration in an open field (**a**). The duration of theta per 5-min blocks was the number of seconds that theta wave activity was observed in that block. Theta peak power was determined with Fast Fourier Transform (FFT) analysis of visually identified theta segments in each 5-min block and normalised to theta peak power of exploratory theta recorded in another room before intraseptal microinjection of bicuculline or vehicle (Veh). In (**b**) intraseptal microinjection of bicuculline significantly increased the ambulatory distance covered in the novel open field. The animal was placed into the open field chamber immediately following intraseptal microinjection. The time course of the effect of drug on ambulation is shown on the left. The histogram plot on the right is the cumulative distance travelled in the first 20 min of exploration in the open field (0–20 min) and the corresponding average speed during ambulation. In (**c**), intraseptal bicuculline significantly enhanced formalin (1.25%, 0.1 ml)-induced ambulation (or agitation). The time course of the effect of drug microinjection on formalin-induced agitation is shown on the left, while phase analysis of the data is shown on right. Formalin was injected at time 0 min immediately following the microinjection of bicuculline or vehicle. Notice the marked cumulative increase in agitation in phase 2 of the formalin test (**c**, right). Interestingly, however, intraseptal bicuculline, did not enhance formalin-induced nociceptive flinching (**d)** that was monitored at the same time as agitation in (**c**). Data are mean ± SEM. Significant difference: (**a**–**c**) *p < 0.05 vs. Veh. Statistical analyses were performed using two-way ANOVA (**a**, **b** [left panel], **c** [left panel]) and unpaired t-test (**b** [right panel], **c** [right panel]).

These experiments were performed so as to investigate the effect of manipulation of intraseptal GABAergic inhibition on septal neural activity and acute nociceptive behaviours. The effect on the former was assessed indirectly using septal-mediated hippocampal theta neural synchronisation as a readout. The GABAergic inhibition was manipulated by intraseptal microinjection of bicuculline, an antagonist at GABA_A_ receptors. Note, that separate group of animals were used for open field (Figs 1 and 2a,b) and formalin experiments (Fig. 2c,d).

#### Effect on hippocampal field activity and ambulation in the open field

Bicuculline (0.125 μg/0.5 μl) microinjected into the MS (e.g. Fig. 1a) immediately before the open field test attenuated the duration of theta wave activity and the FFT theta peak power evoked during exploration in the open field (Figs 1b and 2a). A two-way RM ANOVA showed a significant effect of treatment on both the duration of theta wave activity and the FFT theta power (Fig. 2a; treatment, F_1,8_ > 6.50, p < 0.05). Post-hoc analyses using Bonferroni test for multiple comparisons indicated that bicuculline treatment (n = 6) significantly decreased theta activation for up to 15 min as compared to the vehicle treated animals (n = 4).

On the other hand, bicuculline treatment (n = 7) significantly increased the ambulation in open field as compared to vehicle-treated (n = 6) animals (Fig. 2b; left: treatment, F_1,11_ = 13.44, p < 0.01, two-way RM ANOVA). Post-hoc analyses indicated that bicuculline-induced increase was significant till 15 min post-microinjection (Fig. 2b, left). Indeed, cumulative ambulation was significantly elevated with bicuculline pre-treatment (Fig. 2b, right: t_11_ = 2.80; p < 0.05, unpaired t-test), though the ambulatory speed was unaffected (Fig. 2b, right: t_11_ = 0.095; p > 0.10, unpaired t-test).

#### Effect on formalin-induced nociceptive behaviours

Formalin (0.1 ml, 1.25%) injection into the hind paw evoked a biphasic increase in animal agitation marked by increase in ambulation (Fig. 2c, left), nociceptive licking (data not shown) and flinching (Fig. 2d, left). A two-way RM ANOVA (Fig. 2c, left; treatment, F_1,12_ = 6.70, p < 0.05) followed by post-hoc analyses indicated that bicuculline treatment (n = 7) significantly increased ambulation for up to 20 min as compared to the vehicle-treated animals (n = 7). Phase analysis indicated that the increase was significant in phase 2 (11– 60 min; Fig. 2c, right; t_12_ = 3.62, p < 0.01, unpaired t-test) but not so in phase 1 (0 – 5 min; Fig. 2c, right; t_12_ = 1.95, p > 0.05, unpaired t-test).

On the other hand, bicuculline treatment (n = 7) did not affect formalin-induced licking (data not shown) or flinching (Fig. 2d, right) as compared to vehicle-treated animals (n = 7). The two-way RM ANOVA and unpaired t-test statistics corresponding to flinching are: treatment, F_1,12_ = 1.57, p > 0.05 and, t_12_ = 0.38, p > 0.05 for phase 1 and t_12_ = 1.06, p > 0.05 for phase 2.

### Destructive effect of intraseptal kainic acid on neurons in the medial septal region

**Figure 3:**
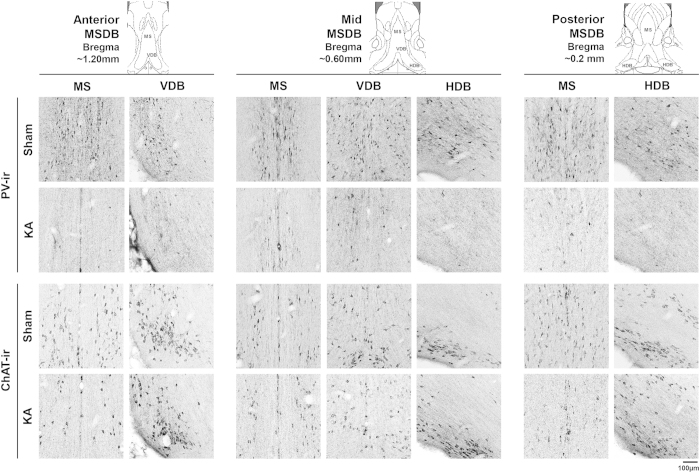
Microinjection of kainic acid into the medial septal region ablates parvalbumin-immunoreactive neurons. Digital images showing parvalbumin (PV)- and choline acetyltransferase (ChAT)-immunoreactive (ir) neurons in coronal sections taken through the medial septum diagonal band region (MSDB). The MSDB is represented by medial septum (MS), vertical limb of the diagonal band of Broca (VDB) and the horizontal limb of the diagonal band of Broca (HDB). Three sets of digital images are shown. The digital images from left to right represent coronal sections taken through the anterior (anterior ~1.20 mm from Bregma), middle (anterior ~0.60 mm from Bregma) and the posterior (anterior ~0.2 mm from Bregma) MSDB. The corresponding diagrammatic representations are shown above each set of images and are adapted from Paxinos and Watson^53^. Sham on the figure are images taken from animals pre-treated with vehicle, while KA on the figure are images taken from animals pre-treated with kainic acid (KA). The PV-ir and ChAT-ir neurons appear as darkly stained against the background. While relatively dense populations of neurons are seen in Sham animals, only neurons labelled for ChAT-ir are observed in sections taken from KA-treated animals.

**Figure 4:**
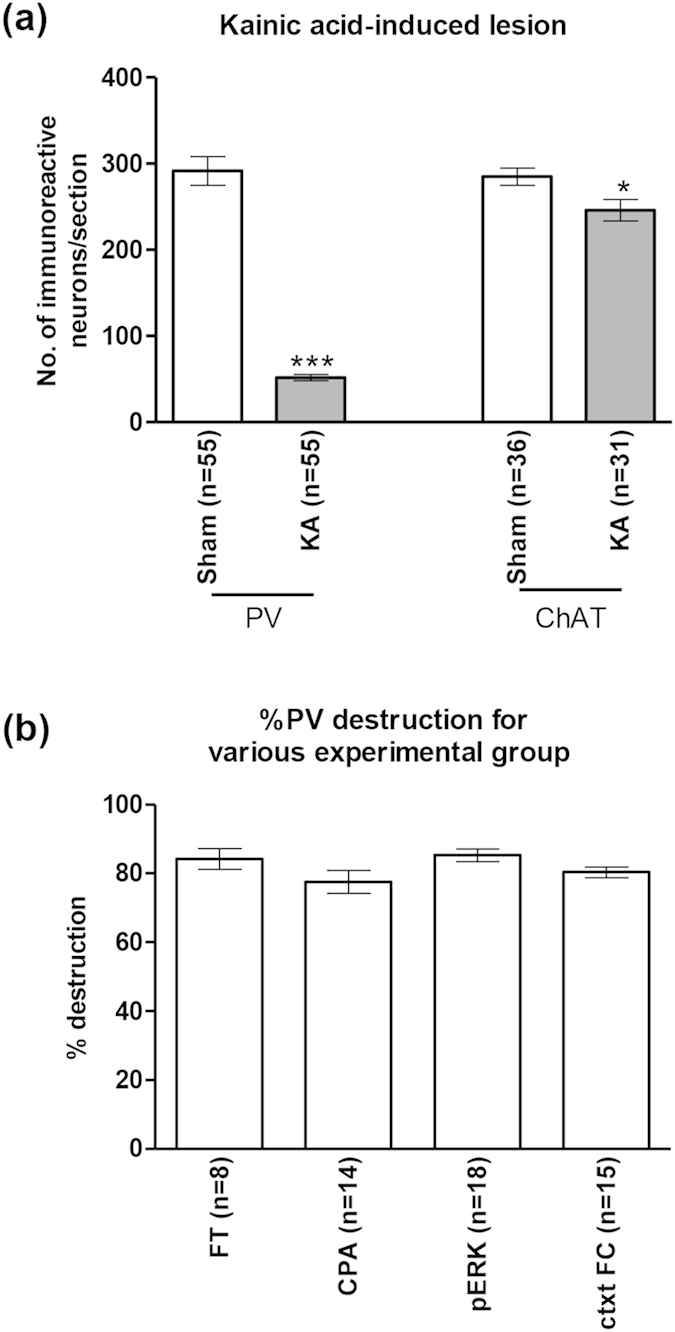
Microinjection of kainic acid into the medial septal region strongly ablates parvalbumin-immunoreactive neurons. Intraseptal treatment with kainic acid (KA) strongly ablated parvalbumin-immunoreactive (PV-ir) neurons in the septal region with a mild effect on the choline acetyltransferase-immunoreactive (ChAT-ir) neurons (**a**). Sham animals were pre-treated with vehicle. The ‘n’ values shown on the figure represent the number of animals in each group. On average, 11 sections were used for counting each marker per animal. (**b**) shows the level of KA-induced destruction of PV-ir neurons in the various experimental groups. The various experimental groups shown are: FT, formalin test; CPA, conditioned place avoidance; pERK, formalin-induced pERK expression in the rACC; ctxtFC, contextual fear conditioning. The ‘n’ values represent the number of animals in each group. Percentage destruction is the number of neurons per section in each KA-treated animal over the average number of neurons per section in the Sham group. Note that the percentage destruction of PV-ir was comparable across the different groups (**b**). Data are mean ± SEM. Statistical significance: (**a**) ***p < 0.001 and *p < 0.05 vs. Sham. Statistical analyses were performed using unpaired t-test (**a**) or one-way ANOVA (**b**).

Intraseptal pre-treatment with kainic acid (KA) evoked a robust and relatively selective destruction of the local parvalbumin (PV)-immunoreactive (ir; putative GABAergic) neurons as compared to the choline acetyltransferase (ChAT; putative cholinergic)-ir neurons in the region (Figs 3 and 4).

The percentage of destruction of PV-ir neurons in MSDB with intraseptal KA was 80.25 + 1.23% (n = 55; range = 70.10–94.70%). On the other hand, the percentage destruction of ChAT-ir neurons was only 19.30 + 3.76% (n = 31; range 0.00–54.66%). The number of PV-ir and ChAT-ir cells in KA lesioned animals were significantly lower than the corresponding values in Sham animals (Fig. 4a; PV: t_108_ = 14.15, p < 0.001, ChAT: t_65_ = 2.47, p < 0.05, unpaired t-test). The destruction of PV-ir and ChAT-ir neurons by KA is comparable to published reports^24^,^25^.

Furthermore, a one-way ANOVA revealed no significant difference in the level of destruction of PV-ir neurons in the various experimental groups (Fig. 4b; groups, F_3,51_ = 2.25, p > 0.05).

### Effect of KA-induced lesion on formalin-induced acute behaviours

These experiments were performed so as to investigate the effect of KA-induced septal lesion on formalin-induced acute nociceptive behaviours.

The effect of KA pre-treatment was examined on animal behaviour to hind paw injection of formalin at two different concentrations, i.e. 1.25% and 2.5%. In general, the lesion had little or no effect on nociceptive behaviours. A weak increase in behaviours was observed in some instances. These results with KA lesion are highlighted below using the experimental group injected with 2.5% formalin. We highlight the results of this group since this concentration was used for studying F-CPA as well.

**Figure 5:**
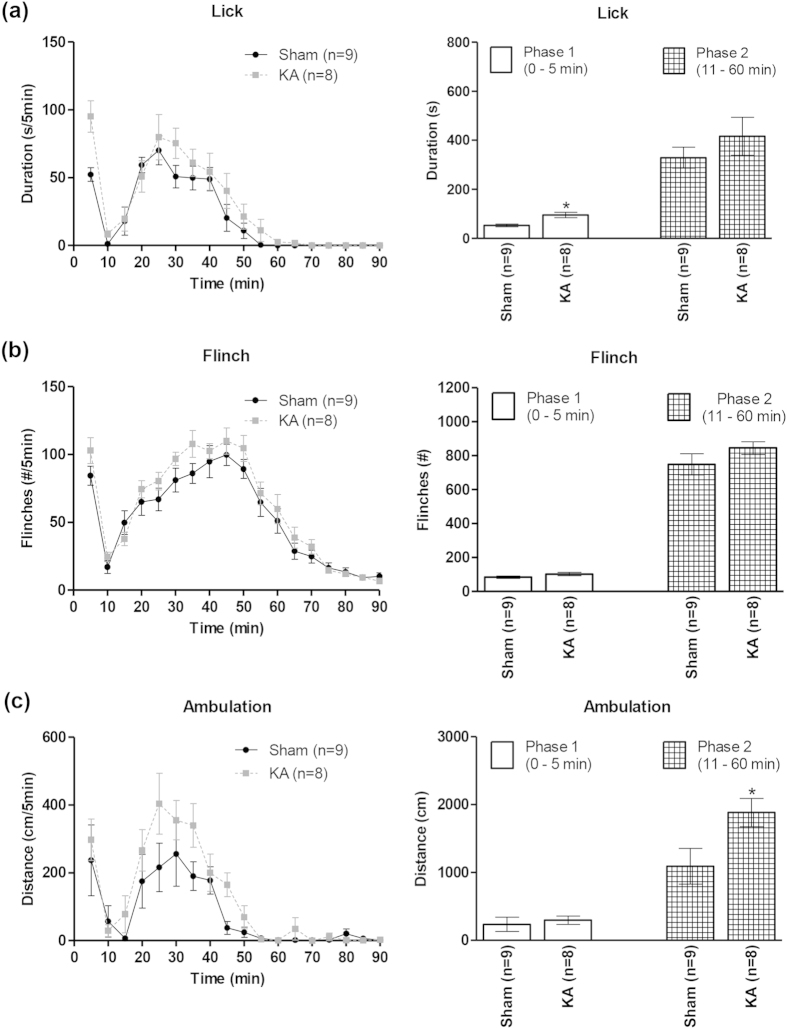
Effect of kainic acid-induced lesion of the septal region on formalin-induced nociceptive behaviours and ambulation. The figure is built as for Fig. 2. Analyses of the time course (left panels) of the formalin-induced licking (**a**), flinching (**b**) and ambulation (**c**), indicated that neuronal destruction with kainic acid (KA) pre-treatment (see Fig. 4) did not significantly alter these behaviours. However, phase analysis (right panels) revealed some changes. For example, licking was increased in phase 1 (0–5 min; (**a**), right). Likewise, an increase in ambulation was observed in the phase 2 (11–60 min; (**c**) right). Data are mean ± SEM. Statistical significance: (a) *p < 0.05 vs. Sham. Statistical analyses were performed using two-way ANOVA (left panels) or unpaired t-test (right panels).

A two-way RM ANOVA showed a lack of effect of KA lesion (n = 8), vis-à-vis sham animals (n = 9), on the time course of formalin (2.5%)-induced licking (Fig. 5a, left; treatment, F_1,15_ = 2.26, p > 0.05), flinching (Fig. 5b, left; treatment, F_1,15_ = 2.08, p > 0.05) and ambulation (Fig. 5c, left; treatment F_1,15_ = 3.53, p > 0.05). Likewise, KA lesion did not affect any of the parameters in animals injected with 1.25% formalin (data not shown).

In line with above, analyses of nociceptive behaviours separated into phase 1 and phase 2 of the formalin test also indicated that KA-induced lesion largely spared nociception. Thus, KA lesion did not affect the total number of formalin-induced flinches in either phase 1 or phase 2 vis-à-vis Sham animals (Fig. 5b, right; t_15_ < 1.59, p > 0.05, unpaired t-test). Similarly, the licking in phase 2 was not affected (Fig. 5a, right; t_15_ = 1.02, p > 0.05, unpaired t-test). However, formalin injection evoked a small increase in licking in phase 1 in KA-treated animals vs. sham animals (Fig. 5a, right; t_15_ = 3.55, p < 0.05, unpaired t-test).

Similar analyses of ambulation revealed that formalin-induced ambulation was significantly increased in phase 2 in KA lesioned animals vs. sham animals in animals injected with 2.5% formalin (Fig. 5c, right; t_15_ = 2.31, p < 0.05, unpaired t-test). The increase in ambulation was much smaller as compared to that with bicuculline (Fig. 2c, right). Ambulation in phase 1 was unaffected with lesion (Fig. 5c, right; t_15_ = 0.49, p > 0.05, unpaired t-test).

### Effect of KA-induced lesion on formalin-induced avoidance

These experiments were performed so as to investigate the effect of KA-induced septal lesion on formalin-induced avoidance, a surrogate measure of affect, in the F-CPA task. The avoidance response was assessed on re-exposing the animal to the conditioning context 24 h after conditioning. Here, it is notable that the lesion did not significantly affect the time course of formalin-induced acute behaviours (see above), thus providing a relatively stable nociceptive and sensorimotor background for analysis of affect. Importantly, lesion had little or no effect on acute nociception.

**Figure 6:**
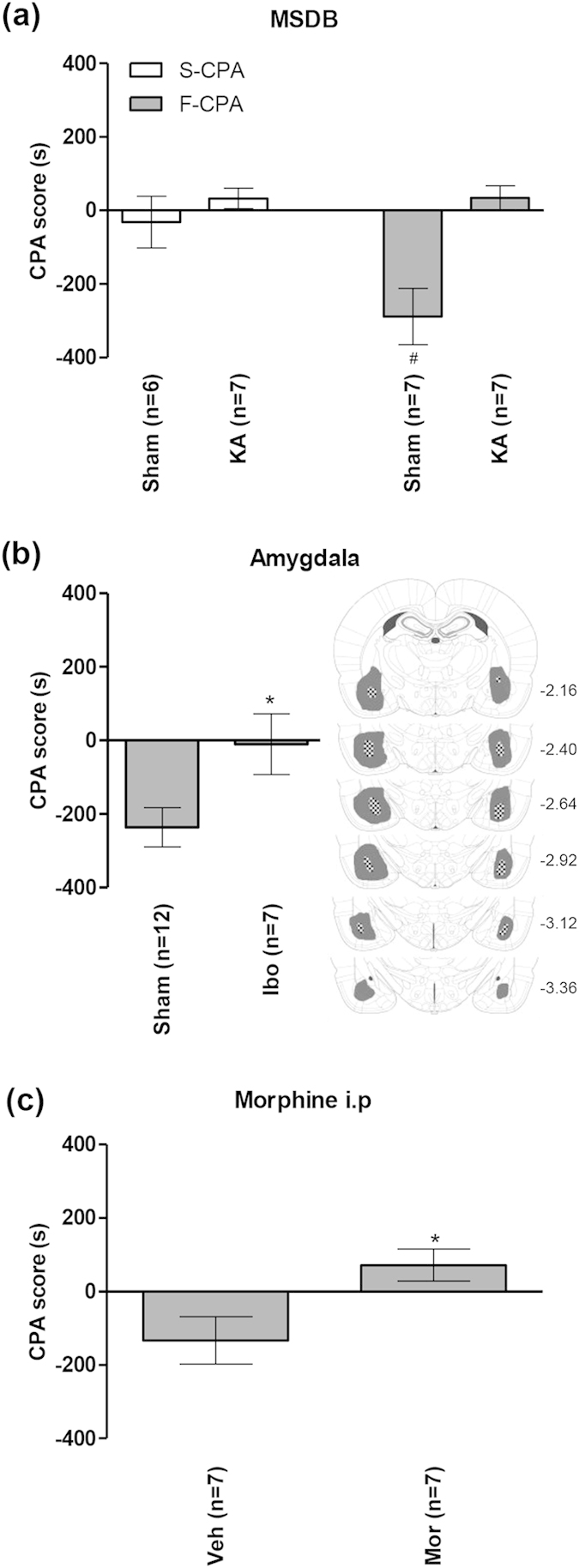
Kainic acid-induced lesion of the medial septal region blocked avoidance learning in formalin induced-conditioned place avoidance task. Avoidance was measured by the CPA score, which is the difference in time spent in the preferred (pain-paired) compartment between test (day 4) and preconditioning (day 2) days of conditioned place avoidance (CPA) training. A negative score indicates avoidance behaviour. In the medial septum diagonal band (‘MSDB’) group (**a**), Sham animals (i.e. vehicle-treated animals) showed avoidance when conditioned with saline-formalin (‘F-CPA’) as compared to animals conditioned with hind paw injection of saline-saline (‘S-CPA’). Neuronal destruction with kainic acid (KA) pre-treatment (see Fig. 4) blocked the formalin-induced avoidance response. In the ‘Amygdala’ group (**b**), bilateral excitotoxic lesion of the amygdala with intra-amygdaloid microinjection of ibotenic acid (Ibo) also eliminated the formalin-induced avoidance response. The diagrammatic representations in (**b**) shows the extent of lesion induced across the anterior-posterior axis of amygdala by ibotenic acid. The shaded and hatched regions represent the largest and the smallest areas of lesion within the amygdala. The number on the right of each diagram represent the anterior-posterior coordinate corresponding to the diagram. In the ‘Morphine i.p’ (intraperitoneal) group (**c**), systemic administration of an anti-nociceptive dose of morphine (5 mg/kg, Mor) abolished the formalin-induced avoidance response compared to that seen in vehicle-treated (Veh) group. Morphine or vehicle were administered just before hind paw injection of formalin during conditioning in the F-CPA experiment. Data are mean ± SEM. Statistical significance: (**a**) ^#^p < 0.05 vs. other groups; (**b**) *p < 0.05 vs. Sham; (**c**) *p < 0.05 vs. Veh. Statistical analyses were performed using one-way ANOVA (**a**) or unpaired t-test (**b**,**c**).

Behavioural analysis indicated that KA-induced lesion of the MSDB region prevented the F-CPA which, otherwise, was quite robust in sham animals (Fig. 6a). A one-way ANOVA revealed significant differences between the groups (Fig. 6a; groups, F_3,23_ = 8.02, p < 0.001). Post-hoc comparisons using the Newman-Keuls test for multiple comparisons revealed that the CPA score of the Sham group conditioned with saline-2.5% formalin (‘Sham F-CPA’ group on Fig. 6a, n = 7) was significantly different from the following control and test groups: Sham and KA-lesioned groups conditioned with saline-saline (‘Sham and KA S-CPA’ groups on Fig. 6a, n = 6 and 7, respectively), and the KA-lesioned group conditioned with saline-2.5% formalin (‘KA F-CPA’ group on Fig. 6a, n = 7). The CPA score for the ‘KA F-CPA’ group was no different from the control groups. KA lesion *per se* did not elicit any place avoidance or place preference on conditioning with saline-formalin or saline-saline as compared to Sham animals conditioned with saline-saline (Fig. 6a).

The effect of KA-induced lesion on F-CPA was similar to that observed with ibotenic acid-induced lesion of the amygdala and intraperitoneal injection of morphine. Thus, bilateral lesion of the amygdala (n = 7) attenuated F-CPA compared to Sham animals (n = 12, Fig. 6b; t_17_ = 2.40, p < 0.05, unpaired t-test). Likewise, intraperitoneal administration of an analgesic dose of morphine^9^ (5 mg/kg, n = 7) also abolished F-CPA vs. animals administered with vehicle (n = 7; Fig. 6c; t_12_ = 2.63 p < 0.05, unpaired t-test). In the F-CPA experiment, morphine (or vehicle) was administered just before hind paw injection of formalin on the day of conditioning.

### Effect of KA-induced lesion on formalin-induced cellular responses in the rostral anterior cingulate cortex

These experiments were performed so as to investigate the effect of KA-induced septal lesion on the expression of pERK in rostral anterior cingulate cortex (rACC) to hind paw injection of formalin (2.5%). No conditioning was involved. The animals were sacrificed 15 min after injection of formalin. Here, it is notable that the formalin-induced unconditioned expression of pERK in rACC is suggested to be a basis of F-CPA^18^.

**Figure 7:**
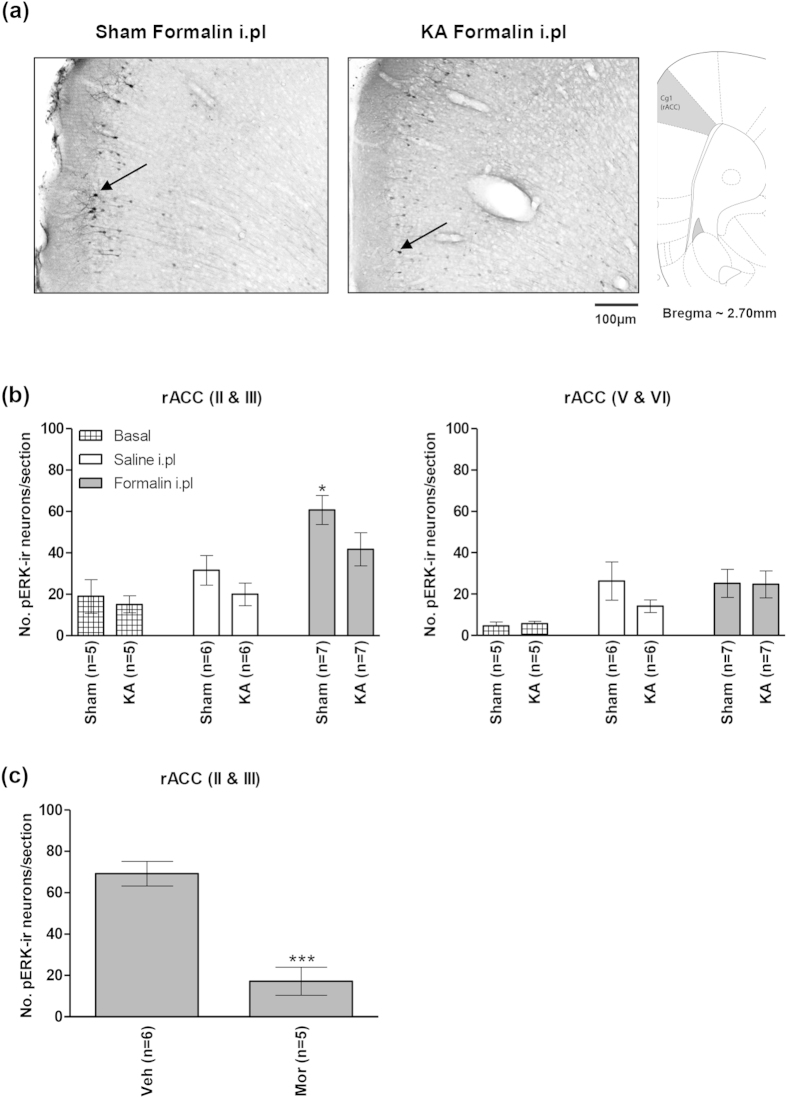
Kainic acid-induced lesion of the medial septal region blocked formalin-induced increase in pERK in the rostral anterior cingulate cortex. At the top are digital representations of sections through the rostral anterior cingulate cortex (rACC; (**a**)). The digital panels show pERK-immunoreactive (pERK-ir) neurons (e.g. those next to arrows in (**a**)) observed on intraplantar (i.pl) injection of 2.5% formalin (‘Formalin i.pl’) into right hind paw of ‘KA’ and ‘Sham’ animals. The ‘KA’ and ‘Sham’ animals were pre-treated with microinjection of kainic acid and vehicle, respectively, into the medial septal region. The effect of KA pre-treatment on populations of neurons in the MSDB is shown in Fig. 4. Note that in the ‘Sham’ group (**b**), hind paw injection of formalin, but not saline (‘Saline i.pl’) evoked a significant increase in number of pERK-ir neurons in layers II and III of rACC that was attenuated with KA pre-treatment ((**b**), left). Hind paw injection of formalin did not evoke a significant increase in the expression of pERK-ir in rACC layers V and VI of the ‘Sham’ group ((**b**) right). In (**c**), pre-treatment with intraperitoneal (i.p) morphine (5 mg/kg, Mor) significantly reduced the formalin-induced pERK-ir in rACC layers II and III as compared to saline-injected controls (Veh). Data are mean ± SEM. Statistical significance: (**b**) rACC (II & III) *p < 0.05 vs. all other groups; (**c**) rACC (II & III) ***p < 0.001 vs. Veh. Statistical analyses were performed using one-way ANOVA (**b**) or unpaired t-test (**c**).

Immunohistochemical analysis showed that KA-induced lesion of the MSDB region attenuated the formalin (2.5%)-induced expression of pERK in layers II & III of rACC which, otherwise, was quite robust in sham animals (Fig. 7a,b). A one-way ANOVA (Fig. 7b, left; groups, F_5,30_ = 6.45, p < 0.001) followed by post-hoc comparisons revealed that the number of pERK-ir neurons in the Sham group injected with intraplantar (i.pl on Fig. 7) formalin (‘Sham Formalin i.pl’ on Fig. 7a, left, n = 7) was significantly higher as compared to the count from other groups, including the KA-lesioned group injected with formalin into hind paw (‘KA Formalin i.pl’ group on Fig. 7b, left, n = 7). The other groups represented on the figure include the following: Sham or KA-lesioned basal (home cage) groups (‘Sham or KA Basal’ on Fig. 7b, left, n = 5 each), and Sham or KA-lesioned animals injected with saline into hind paw (control groups; ‘Sham or KA Saline i.pl’ on Fig. 7b, left, n = 6 each). The home cage groups, the control groups and the KA Formalin i.pl group were not different from each other. The lack of effect of KA-induced lesion on the expression of pERK in home cage groups and control groups suggests that the lesion *per se* do not affect the basal levels of pERK-ir in rACC.

In addition to above, we also counted the number of pERK-ir cells in layers V & VI of the rACC (Fig. 7b, right). We separated the count from these deeper layers from the count in the superficial layers II & III since the effect of formalin on pERK expression in the deeper layers is variable^18^,^26^. In the present study, a one-way ANOVA revealed significant difference between groups (Fig. 7b, right; groups, F_5,30_ = 2.54, p < 0.05). In this regard, injection (be it saline or formalin) alone tended to increased pERK in the region as compared to basal (home cage) groups. However, post-hoc comparison indicated that the individual counts did not reach the accepted level of significance.

Finally, an attenuation of the formalin-induced expression of pERK in layers II & III of the rACC was also seen with intraperitoneal administration of the analgesic morphine as compared to vehicle-injected animals (5 mg/kg, Fig. 7c; t_9_ = 5.81, p < 0.001, unpaired t-test).

### Effects of KA-induced lesion on contextual fear

These experiments were performed so as to determine the effect of KA-induced septal lesion on fear evoked on conditioning with relatively mild noxious/aversive stimuli. Animal freezing on re-exposure to context 24 h after conditioning (i.e. retrieval) was taken as index of fear.

**Figure 8:**
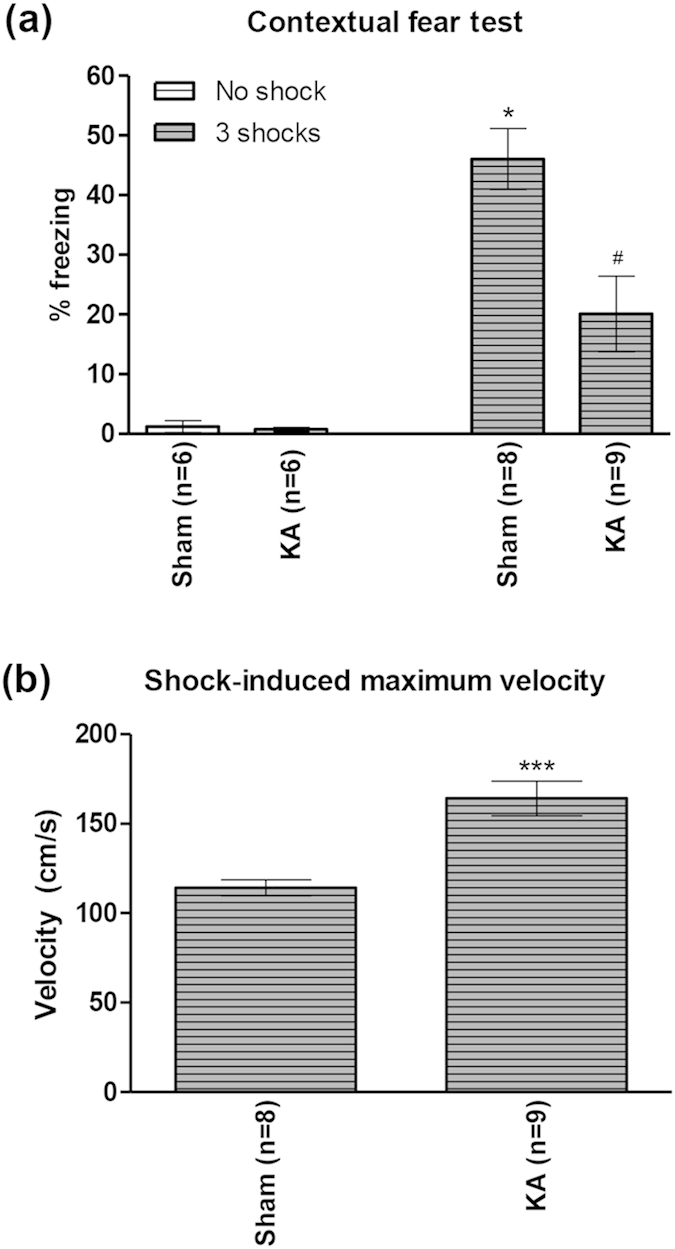
Kainic acid-induced lesion of the medial septal region attenuated contextual fear. In (**a**), freezing response was reduced in ‘KA’ group vs. ‘Sham’ group. ‘KA’ and ‘Sham’ groups were pre-treated with microinjection of kainic acid and vehicle, respectively, into the medial septal region. The effect of KA pre-treatment on populations of neurons in the MSDB is shown in Fig. 4. The different groups of animals were either conditioned with 3 footshocks (‘3 shocks’ on the figure) or did not receive any shock (‘No shock’ on the figure). Interestingly, KA pre-treatment did not attenuate acute reaction to footshock. Indeed, acute reaction, as measured by maximum velocity recorded during footshock, was enhanced in ‘KA’ group as compared to ‘Sham’ group (**b**). Data are mean ± SEM. Statistical significance: (**a**) *p < 0.05 vs. other groups; ^#^p < 0.05 vs. Sham No shock and KA No shock; (**b**) ***p < 0.001 vs. Sham. Statistical analyses were performed using one-way ANOVA (**a**) or unpaired t-test (**b**).

Behavioural analysis indicated that KA-induced lesion attenuated freezing as compared to sham animals (Fig. 8a). A one-way ANOVA (Fig. 8a; groups, F_3,25_ = 18.7, p < 0.0001) followed by post-hoc comparison indicated that the freezing in ‘Sham 3 shocks’ group (n = 8) was significantly higher than other groups, including the ‘KA 3 shocks’ group (n = 9; Fig. 8a). The freezing behaviour in the ‘KA 3 shocks’ group, while significantly lower than ‘Sham 3 shocks’ group was higher than that in the ‘No shock’ groups (n = 6 each). The effect of KA-induced lesion on freezing was observed in absence of an attenuation of footshock-induced sensory responses. Rather, the maximum velocity recorded during footshocks was enhanced (Fig. 8b; t_15_ = 4.55, p < 0.001, unpaired t-test).

### Effects of KA-induced lesion on conditioned cellular responses

These investigations were performed so as to determine the effect of KA-induced septal lesion on the conditioned response-induced expression of Egr-1/Zif268 in the amygdala and the hippocampus. The cellular responses were analysed using brain tissues taken from animals used in experiments illustrated above. Here, to reiterate that both amygdala and hippocampus show fear-induced conditioned expression of Egr-1/Zif268^19^, though little is known of the conditioned cellular responses in the two regions in the F-CPA task.

**Figure 9:**
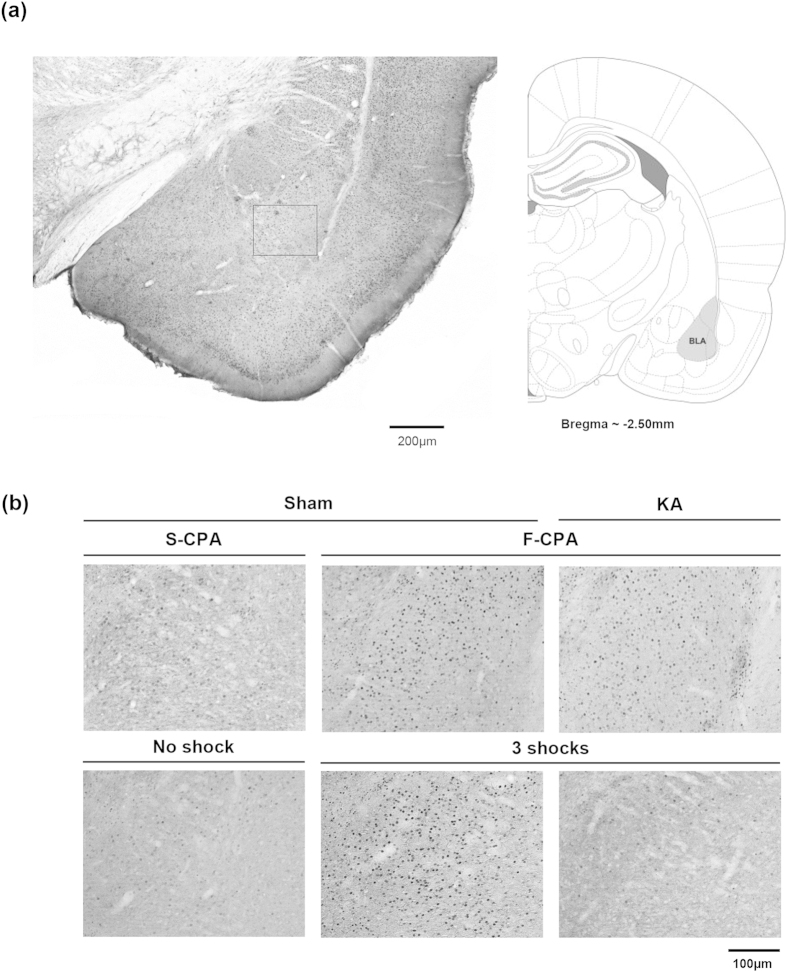
Egr-1/Zif268 was induced in the basolateral amygdala during conditioned place avoidance and contextual fear. ((**a**) left panel) is digital image of a Egr-1/Zif268-immunolabelled coronal section taken through the basolateral amygdala (BLA). The corresponding diagrammatic representation and the related anterior-posterior coordinate are on right (**a**). The diagrammatic representation is adapted from Paxinos and Watson^53^. (**b**) are digital images showing Egr-1/Zif268-immunoreactivity (ir) in the area of BLA that corresponds to the boxed region shown in (**a**). Nuclei of cells containing Egr-1/Zif268-ir stand out darkly stained relative to the background on the digital images. The sections in (**b**) are taken from ‘Sham’ and ‘KA’ animals that were pre-treated with microinjection of vehicle and kainic acid, respectively. The sections are from animals used to develop Fig. 10. The effect of KA pre-treatment on population of neurons in the MSDB is shown in Fig. 4. The treated animals were either (i) conditioned with saline-saline (‘S-CPA’ or saline-conditioned place avoidance) or saline-formalin (‘F-CPA’ or formalin-conditioned place avoidance), or (ii) conditioned with footshocks (‘3 shocks’) in the contextual fear task. Animals that did not receive any footshock (‘No shock’ on figure) acted as controls for the ‘3 shocks’ group. Notice the relatively high number of Egr-1/Zif268-ir cells in the sections taken from ‘Sham F-CPA’ and ‘Sham 3 Shocks’ animals ((**b**) middle panels) as compared to the corresponding control ((**b**) left panels). On the other hand, little or no Egr-1/Zif268-ir was observed in ‘3 shocks’ animal following pre-treatment with KA ((**b**) lower row, right panel).

**Figure 10:**
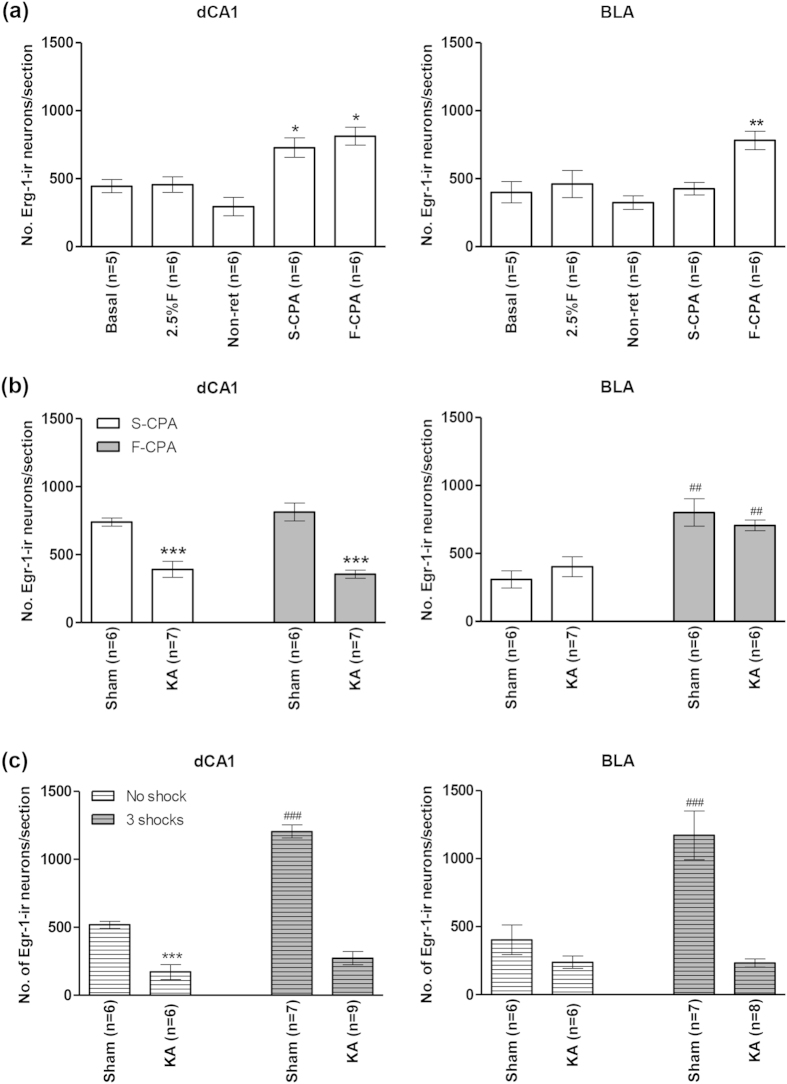
Kainic acid-induced lesion attenuated the contextual fear- but not avoidance-induced expression of Egr-1/Zif268 in basolateral amygdala. In (**a**), the effect of conditioning with saline-saline (S-CPA or saline-conditioned place avoidance) or saline-formalin (F-CPA or formalin-conditioned place avoidance) on expression of Egr-1/Zif268-immunoreactivity (ir) was compared vis-à-vis the following groups of animals: home cage (*Basal*); injected with 2.5% formalin into hind paw and sacrificed 2 h later (*2.5% F*); conditioned with saline-formalin but without being re-exposed to the training apparatus on the test day (*Non-ret*). All the animals were Sham animals, that is, they were pre-treated with microinjection of vehicle into the medial septum diagonal band (MSDB). Notice that the number of Egr-1/Zif268-ir neurons were increased in dorsal hippocampus field CA1 (dCA1) to a similar extent in the S-CPA and F-CPA groups of animals. However, in the basolateral amygdala (BLA), the protein was induced only in the F-CPA group. In (**b)**, the effects of conditioning (S-CPA and F-CPA) were compared in kainic acid (KA)-pre-treated animals vs. Sham animals. The neuronal destruction with KA pre-treatment is shown in Fig. 4. The lesion prevented the conditioning-induced expression of Egr-1/Zif268 in dCA1. However, the destruction did not prevent the selective increase of Egr-1/Zif268 in BLA in the F-CPA group. In (**c**), KA pre-treatment prevented the contextual fear-induced increase of Egr-1/Zif268 in both dCA1 and BLA. Data are mean ± SEM. Statistical significance: ((**a**) dCA1) *p < 0.05, vs. Basal, 2.5%F and Non-ret; ((**a**) BLA) **p < 0.01, vs. other groups; ((**b**) dCA1) ***p < 0.001, vs. Sham S-CPA and Sham F-CPA; ((**b**) BLA) ^##^p < 0.01, vs. Sham S-CPA and KA S-CPA; ((**c**) dCA1) ***p < 0.001 vs. Sham No shock; ^###^p < 0.001 vs. other groups; ((**c**) BLA) ^###^p < 0.0001 vs. other groups. Statistical analyses were performed using one-way ANOVA.

Immunohistochemical analyses showed that the number of Egr-1/Zif268-ir neurons increased in BLA (Figs 9 and 10a, right) and dorsal CA1 (dCA1; Fig. 10a, left) in the F-CPA task. The increase in dCA1 was related to the experimental context and not to an effect of injection of formalin. Thus, a one-way ANOVA (groups, F_4,24_ = 11.8, p < 0.0001) followed by post-hoc comparisons showed that the numbers of Egr-1/Zif268-ir cells were increased in dCA1 on the test day in non-lesioned animals conditioned with saline-saline (S-CPA, n = 6) or saline-2.5% formalin (F-CPA, n = 6) vs. the levels observed in the various control groups (n = 5 or 6 each, Fig. 10a, left). On the other hand, the increase in BLA was related to conditioning with formalin. Thus, a one-way ANOVA (groups, F_4,24_ = 6.44, p < 0.01) followed by post-hoc comparisons showed that an increase in number of Egr-1/Zif268-ir cells in BLA was observed only in animals conditioned with saline-2.5% formalin (F-CPA, Fig. 10a, right).

Interestingly, KA-induced lesion prevented the context-related increase in dCA1 (Fig. 10b), but not the aversion-related increase in BLA (Fig. 10b). In context of the former, a one-way ANOVA (groups, F_3,22_ = 22.7, p < 0.0001) followed by post-hoc comparisons indicated that the number of Egr-1/Zif268-ir cells in dCA1 in KA-lesioned animals in the F-CPA task (‘KA F-CPA’ on Fig. 10b, left; n = 7) or the S-CPA task (‘KA S-CPA’ on Fig. 10b, left; n = 7) were significantly lower than the corresponding values in the sham animals. As regards to the BLA, a one-way ANOVA (groups, F_3,21_ = 10.3, p < 0.001) followed by post-hoc comparisons indicated that the number of Egr-1/Zif268-ir cells in BLA in KA-lesioned animals in the F-CPA task (‘KA F-CPA’ on Fig. 10b, right; n = 6) was comparable to the corresponding value in the Sham animals (‘Sham F-CPA’ on Fig. 10b, right; n = 6), while both were higher than the control groups (n = 6 or 7).

As regards to contextual fear, the KA-induced lesion prevented the expression of Egr-1/Zif268-ir in both dCA1 and BLA (Fig. 10c). A one-way ANOVA showed significant differences between groups in both dCA1 (Fig. 10c, left; groups, F_3,24_ = 94.4, p < 0.0001) and BLA (Fig. 10c, right; groups, F_3,23_ = 15.2, p < 0.0001). Post-hoc comparisons indicated that the number of Egr-1/Zif268-ir neurons in both dCA1 and BLA were significantly higher in sham animals conditioned with footshocks (‘Sham 3 shocks’ on Fig. 10c; n = 8 each for dCA1 and BLA) vs. KA-lesioned animals conditioned with footshock (‘KA 3 shocks’ on Fig. 10c; n = 9 each for dCA1 and BLA). Comparison of ‘Sham No shock’ animals vs. ‘KA No shocks’ (n = 6 each) indicated that the lesion attenuated expression of Egr-1/Zif268 in dCA1, but not BLA, on exposure to context alone, an effect similar to that seen in the F-CPA task (Fig. 10b, see above).

## Discussion

The present study, juxtaposed with our published data, provides evidence which suggests that the medial septum is a nodal centre in the forebrain that modulates a range of behaviours to noxious-aversive stimuli. In this context, we have previously shown that the MSDB facilitates acute nociceptive behaviours and spinal nociceptive transmission in the formalin model of persistent inflammatory pain^9^. In the present study, we report the novel findings that KA-induced lesion of the MSDB region abolished the expression of formalin-induced affective behaviour, which was assessed using the F-CPA task. Such destruction, however, evoked little or no effect on acute nociception. Indeed, only licking behaviour was affected and that too only in phase 1 of the formalin test. The nociceptive behaviours were also not affected with intraseptal microinjection of the GABA_A_ receptor antagonist, bicuculline. In addition, we found that the MSDB facilitated affective responses to a range of noxious stimuli since KA-induced lesion of the MSDB also prevented the expression of contextual freezing to brief footshock.

Intriguingly, both intraseptal bicuculline and KA-induced lesion enhanced formalin-induced agitation, which was measured as ambulation. Broadly, this is consistent with the postulate that the region, especially the septo-hippocampal network, modulates sensorimotor behaviours^17^,^27^,^28^,^29^. At present, the neural basis of the sensorimotor effects of the two treatments in the formalin test remains unclear. However, analysis of the present and published evidence suggests the two treatments affect the septo-hippocampal theta network. In this context, intraseptal bicuculline attenuated septo-hippocampal theta activation (present study), while others have reported a similar effect of KA-induced lesion of MSDB^24^,^25^. In the latter study, the level of destruction of PV-ir neurons was similar to that observed in the present study. Besides, KA also destroy a population of non-PV-ir, intraseptal GABAergic neurons^24^. Whereas, the septo-hippocampal theta generation is postulated to involve GABA-mediated rhythmic intraseptal inhibition and a rhythmic output, especially via PV-ir projection neurons, to the hippocampus^25^,^27^,^28^,^29^. The attenuation of theta by bicuculline in the present study also reflects a pharmacological impairment of theta generation and is not secondary to behavioural changes since the attenuation was accompanied by an increase, and not decrease in animal ambulation. Indeed, theta activation in rodent is most robust with exploration, including during formalin-induced agitation in rodent^16^,^17^.

However, intraseptal bicuculline, unlike KA-induced destruction is unlikely to attenuate efferent signal from MSDB, especially given that it attenuates local inhibition. Intriguingly, the PV-ir septo-hippocampal neurons are active during locomotion^30^. Collectively, the preceding raises a possibility that sensorimotor effect of the two treatments in the formalin test is driven, in part, by an impairment of intraseptal inhibition which, in turn, facilitates stronger neural excitation during ambulation. Stronger efferent signal from MSDB, carried partly by PV-ir neurons, enhances ambulation. In this scenario, the weaker effect on formalin-induced ambulation with KA lesion, as compared to intraseptal bicuculline, arises in part due the destruction of MSDB efferent, including the PV-ir neurons.

Importantly, however, the sensorimotor effects in the formalin-test are dissociated from nociception. Indeed, while intraseptal bicuculline-induced agitation in the formalin test is sustained during the inter-phase of the formalin test, the nociception is at its lowest during this period. Furthermore, intraseptal bicuculline and KA lesion of MSDB increased agitation in phase 2 of the formalin test without significantly affecting nociceptive licking and flinching during that period.

Interestingly, the finding that intraseptal bicuculline does not attenuate acute nociception, although it attenuates theta activation, suggests that the generation of theta-rhythmic neural activity in phase 1 of the formalin test is not crucial for acute nociception. On the other hand, we have previously shown that the inhibition of neural activity in MSDB in the first phase of the formalin test attenuates acute nociception in the second phase of the formalin test^9^. Put together, the foregoing suggests that the non-theta-related neural activation of MSDB in the first phase plays a more crucial in modulating acute nociceptive behaviours. Furthermore, the present data also indicate that the septal PV-ir GABAergic efferent are also not crucial for formalin-induced acute nociception since the destruction of these neurons evoked little or no effect on formalin-induced acute nociceptive behaviours. Though, the destruction sufficed to attenuate animal avoidance behaviour in the F-CPA task. Collectively, the foregoing suggests that the septal mechanisms mediating acute nociception in the formalin test are separate from those that underpin aversive learning to formalin-pain.

In context of F-CPA, the effect of lesion on aversion was comparable to the effects seen with administration of a moderate dose of the prototypical narcotic analgesic, morphine and the bilateral lesion of amygdala, a region known to facilitate F-CPA^3^,^31^. The lesion also attenuated contextual fear induced on conditioning with footshock. Interestingly, cellular analysis in the current study indicated that the KA lesion attenuated formalin-induced unconditioned expression of pERK in layers II & III of the rACC. This effect of lesion mimicked the change observed with the analgesic morphine. Additionally, others have reported that the level of pERK in rACC also increases on expression of avoidance behaviour in the F-CPA task^18^. Both unconditioned and conditioned expression of pERK in rACC is implicated in formalin aversion but not acute behaviours^18^,^32^. Notably, the expression of pERK in layers II & III of the rACC is linked with long-term potentiation of synaptic transmission (LTP) in the region, LTP being a potential cellular mechanism of learning and memory and also a cellular basis of chronic pain^26^,^33^,^34^,^35^. Moreover, the ACC modulates the acquisition of both ctxtFC and F-CPA by mechanisms that overlap, at least in part, and involve LTP-like changes that are mediated partly by the NMDA receptors^36^,^37^,^38^,^39^.

Collectively, the preceding suggests that the MSDB is a nodal centre wherein neurons, including GABAergic neurons, mediate nociceptive affect-motivation, in part, by regulating cellular mechanisms in ACC that confer an aversive value to the noxious stimulus. However, the lesion evoked a variable effect on conditioned responses in amygdala and hippocampus. Thus, while the lesion attenuated the conditioned increase in Egr-1/Zif268 evoked in CA1 and BLA in parallel with loss of fear in the ctxtFC task, it did not attenuate the formalin conditioning-induced increase in Egr-1/Zif268 in BLA. The increase in Egr-1/Zif268 in dorsal CA1 and BLA in contextual fear has been reported before and, indeed, Egr-1/Zif268 in dorsal CA1 and BLA has been linked to re-consolidation and acquisition of fear, respectively^19^,^20^,^21^. Whereas, in context of F-CPA, we show that conditioning with formalin evoked an increase in BLA but not dorsal CA1.

The generation of conditioned responses in lesioned animals in the F-CPA task might reflect the strength and/or the persistent nature of nociception with formalin vis-à-vis the brief footshock in the ctxtFC task. Indeed, there was no obvious change in animal behaviour with the lesion that might suggest that the expression of Egr-1 in lesioned animals reflected an emergence of a novel motivational state. In this regard, the lesion did not lead to anti-nociception or an altered place preference behaviour. Presumably, however, the formalin conditioning-induced response in BLA, in absence of septal-mediated aversive representation in cortex, is dissociated from aversive value of formalin-pain in lesioned animals.

In addition to the effect on nociceptive processing in rACC, KA-induced lesion in the current study also attenuated the conditioned representation of experimental context in dorsal hippocampal field CA1 in the F-CPA task. Indeed, hippocampal neurons process contextual/spatial information, while the septal GABAergic mechanisms are implicated in the mediation of hippocampal-dependent spatial memory tasks^40^,^41^,^42^. The preceding, seen in conjunction with the effects of the lesion on rACC and behaviour, suggests that the MSDB may integrate affect with context as part of associative learning in the F-CPA task.

The network and cellular mechanisms underlying the cellular and behavioural effects of KA-induced destruction remain unknown. It is unclear whether the septal PV-ir neurons project to the rACC, although they project extensively to the hippocampus^43^. Whereas, the septal cholinergic neurons, which were also somewhat destroyed by KA, project to the rACC^8^. Indeed, the MSDB cholinergic neurons are implicated in nociceptive processing and in mediation of contextual fear^44^,^45^. Moreover, the septal neurons, including GABAergic, cholinergic and glutamatergic form an interconnected network^46^,^47^. As a result, disrupting a neuronal population, for example the GABAergic neurons, may affect the output from other projection neurons in the region, including the cholinergic neurons^41^. Therefore, KA lesion may evoke a direct and/or an indirect effect on septal output to affect processing in hippocampus and rACC.

Collectively, the present findings suggest that the GABAergic neurons of the MSDB region facilitate aversion. From a broader perspective, the findings position the MSDB region as a substrate of the reward-aversion circuitry that is postulated to sustain neural changes mediating the aversive-fearful state of pain^48^,^49^,^50^,^51^,^52^.

## Methods

### Animals and general procedure

Adult male Sprague-Dawley rats (270–320 g at the time of surgery) were used for all experiments. The experiments described in this manuscript followed the ethical guidelines of the International Association for the Study of Pain and were approved by the local Institutional Animal Care and Use Committee of the National University of Singapore.

### Survival surgery

Survival surgery was performed on rats so as to microinject neurotoxins (kainic acid or ibotenic acid) into region of interest or implant recording electrodes and/or microinjection cannulas in the brain tissue. Rats were either anesthetised with sodium pentobarbitone (60 mg/kg) or an isoflurane-oxygen mixture (5% for induction and 1.5–2% for maintenance) and mounted onto a stereotaxic frame with the surface of the skull in a horizontal plane. Incisions were made along midline and burr holes were then drilled on the exposed skull at select stereotaxic coordinates according to Paxinos and Watson^53^. The surgical procedures performed on the animals are as described below.

#### Implantation of microinjection cannula and or electrode

A single 26G microinjection stainless steel guide cannula (Plastic One) was implanted into the MSDB (AP 0.5 mm, ML 0.0 mm, DV 5.4 mm from the skull surface) and subsequently secured to the skull with stainless screws and dental cement (Shofu Inc., Kyoto, Japan). In some animals, depth recording electrodes were implanted together with microinjection cannula, to investigate the effect of drug microinjection on hippocampal field activity. The electrodes were constructed by twisting together a pair of stainless steel wires (A-M system, Carlsborg, WA, USA) and implanted into the dorsal hippocampal field CA1 (AP −4.0 mm, ML 2.2 mm on the left from the midline, DV 3.2 mm from the skull surface). Each wire was approximately 0.125 mm in diameter and 40 mm in length. The wires were insulated with Teflon except for the tips. The two tips of the electrode that were lowered into the brain were spaced 0.5–1 mm apart in vertical direction. Separate gold-plated male connector pins (A–M Systems) were soldered onto the other end of the wires. The screw electrode, used as a ground electrode, consisted of miniature stainless steel bone screw (Fine Science Tools Inc., North Vancouver, BC, Canada) soldered to a 40 mm long Teflon-insulated silver wire of approximately 0.25 mm in diameter (A–M Systems). The free end of the silver wire was soldered onto a gold-plated male connector pin (A–M System). Once the recording electrode was lowered to its desired position, it was secured to an anchor screw with dental cement, the male connector pins of all the electrodes were pushed into a 9-pin ABS plug (Ginder Scientific, Nepean, ON, Canada) which was then secured onto the skull with dental cement.

#### Microinjection of neurotoxins

A 33G, stainless steel microinjection needle, attached to a 5-μl Exmire microsyringe (Ito Corporation, Japan) was directed into either the amygdala (AP −2.5 mm, ML ± 4.8 mm, DV 8.5 mm from the skull surface) or the MSDB (AP 0.5 mm, ML 0.0 mm, DV 6.5 mm from the skull surface). Ibotenic acid (10 μg/μl; or vehicle) was injected bilaterally (0.3 μl per side) into the amygdala. Ibotenic acid, rather than kainic acid was used to lesion amygdala so as to replicate published observations. In this regard, Tanimoto *et al.* have reported that ibotenic acid-induced lesion of BLA attenuates avoidance behaviour in the F-CPA task^3^. In the current study, the experiments involving amygdaloid lesion served as a comparison for the effect of kainic acid-induced lesion of the MSDB region.

In the MSDB, a total volume of 0.5 μl of kainic acid (1 μg/μl; or vehicle) was injected into the midline of the region. Drugs were administered over a 5-min period. The microinjection needle was left in place for an additional 10 min after the last injection to allow the drug solution to diffuse to the surrounding tissue. Subsequently, the incision site was sutured with non-absorbable polyamide suture (metric size 4.0, B.Braun, Aesculap Inc., USA).

### Behavioural experiments

In general, behavioural experiments were performed between 10 and 14 days after microinjection of neurotoxins or implantation of cannula. Animals were habituated to the experimenter and the behavioural equipment in experiments involving injections of formalin (see below), while non-habituated animals were used for the remaining behavioural experiments. It should also be noted that experimenters performing behavioural tests were blinded to the drug treatment.

#### Open field exploration and hippocampal EEG recording

Hippocampal field activity during exploratory behaviour was recorded via a thin flexible wire screwed on to the animal’s head stage. Theta wave activity was first recorded for at least 1 min during animal exploration in a laboratory adjacent to the behavioural suite. Following that, the animal was transferred to a novel room in the behavioural suite for the open field experiment. Bicuculline (0.125 μg/0.5 μl) or vehicle (0.5% w/v alcian blue saline) was microinjected into the MSDB. The dose of bicuculline used in the present study is at the lower end of the dose-range used in literature^54^. At the selected dose, intraseptal bicuculline did not evoke any seizure-like behaviour or epileptic-like hippocampal after-discharges^54^ (present study). A volume of 0.5 μl of solution was microinjected over a span of 30 s via the internal cannula. Following microinjection, the internal cannula was left in place for an additional 1 min to allow the drug diffusion before its removal. Checks were performed by pushing out some solution just before insertion of the internal cannula and also on removal of the internal cannula after microinjection so as to ensure that microinjection assembly system was functioning properly. After microinjection, the animal was immediately placed at the lower left corner of an open field activity monitor (43.2 cm × 43.2 cm × 30.5 cm, L × W × H; Model ENV-515, Med Associates Inc., USA) for 60 min. A continuous recording of hippocampal field activity was also made during this 60-min period. Animal’s movements and position were tracked by the open field monitor using 3 sets of 16 evenly spaced infrared (IR) transmitters and receivers positioned along the perimeter of the chamber. An ambulatory episode was signalled when the rat moved beyond four in-built infrared beams (i.e. box size of about 10 × 10 cm) in 500 ms. The average speed of movement was calculated by dividing the distance moved by the time taken to cover that distance.

#### Formalin test

The formalin test was performed in a clear plastic observation chamber (43 × 21.6 × 30 cm, L × W × H) of an open field activity monitor (model ENV-515, Med Associates Inc.). Prior to the day of formalin test, animals were habituated to the experimental chamber for at least 45 min each day for 3d. On the day of formalin test, animals were generally habituated for at least 15 min before hind paw injection of formalin. For experiments involving drug microinjection into the MSDB, bicuculline (0.125 μg/0.5 μl) or vehicle (0.5% alcian blue saline) was first microinjected into the MSDB. After removal of the internal cannula, formalin (1.25%) was injected into the right hind paw while the animal was gently restrained. For experiments involving lesioned animals, the procedure was identical except without drug microinjection. In addition, formalin concentration of either 1.25% or 2.5% was injected into the right hind paw.

Formalin-induced nociceptive behaviours were monitored by the same experimenter for a period of 90 min after hind paw injection of the algogen. Licking and flinching of the injected paw were taken as behavioural indices of formalin nociception. Licking was measured as the time spent licking the injured paw at both the dorsal and plantar surfaces. Flinching was the number of shakes and jerks of the injured hind paw. In addition, formalin-induced moment-to-moment agitation was also measured by recording animal ambulatory movements using the open field activity monitor (see above).

#### Conditioned place aversion (CPA)

The place conditioning apparatus was a clear acrylic box (46 × 46 × 40 cm) divided into two equal-sized compartments. The two compartments were separated by a black wall with an opening (10 × 46 cm) in the centre to allow access between both compartments. An additional sliding door was available to close off the opening so as to confine the rat to a given compartment during conditioning. The apparatus was designed such that each compartment had distinct visual and tactile cues. In this regard, the walls of one the compartment were white, while the floor was textured. Whereas, the walls of the adjoining compartment was either black (long wall) or black with white vertical strips (short walls) while the floor was smooth. The apparatus was cleaned thoroughly with 70% ethanol each time after use.

CPA experiments were conducted using a four-day biased protocol as described previously^3^,^55^. Briefly, on the days 1 and 2, the sliding door was removed and the animal was allowed to explore both compartments freely for 900 s. The time spent in each compartment was recorded automatically by an activity monitoring software (ActiMot, version 6.08, TSE Systems, GmbH, Germany). The preference of the animal was noted and a biased F-CPA design was used, i.e. formalin injection was paired with the preferred compartment. Only animals which showed a consistent preference for the same compartment across both days were used for the study. In addition, animals were excluded from the study if they showed (i) extreme preference i.e. spent more than 80% of the time (720 s) in one compartment on either day or (ii) a difference of more than 200 s in the same compartment between day 1 and day 2.

On day 3, place conditioning took place in two sessions. In the morning, the animal was given an intraplantar (i.pl) injection of saline (0.1 ml) into the left hind paw and confined to its non-preferred compartment for 1 h. At least 3 h later, the same rat was given an i.pl injection of formalin (2.5%, 0.1 ml) into the right hind paw and confined to its preferred for 1 h (F-CPA). The concentration of formalin was based on a pilot study where injection of 2.5% formalin produced a robust avoidance behaviour as compared to injection of 1.25% formalin. Some animals received injection of saline for both conditioning sessions (hence S-CPA) and acted as controls.

On day 4, each rat was started off in the non-preferred compartment and allowed to explore the apparatus freely for 900s. Avoidance behaviour was measured by the CPA score, which is the difference in time spent in the preferred-compartment between day 4 (test day) and day 2 (preconditioning day).

In experiments which investigated the effect of systemic morphine on F-CPA, the experiment was conducted as described above with some differences on the conditioning day (day 3). In this regard, for the first conditioning session, the animal received an intraperitoneal (i.p) injection of saline (0.1 ml/kg) followed immediately by i.pl injection of saline into the left hind paw. For the second conditioning session, the animal received either an i.p injection of morphine (5 mg/kg) or saline (as control) followed immediately by i.pl injection of formalin (2.5%) into the right hind paw.

#### Contextual fear conditioning (ctxtFC)

Contextual fear training and recall were performed in a one-chamber fear conditioning system (V8.04, TSE Systems, Germany). The system consisted of an acrylic training chamber (45 × 45 × 50 cm) placed in a sound-attenuating housing. A video camera was positioned on the top of the housing to allow subjects’ behaviour to be observed and recorded by an experimenter. The floor of the chamber consisted of 25 stainless steel grid rods (diameter 4 mm) spaced 1 cm apart (centre-to-centre) wired to a shock generator for the delivery of footshock. The training context was enhanced to provide additional visual cues. These visual enhancements included black and white vertical stripes and bright coloured circles on a black background on the right and left side of the conditioning chamber, respectively. Throughout contextual fear training and testing, the lights in the experimental room were switched off. The only illumination was provided by the housing lights and was maintained at 150 lux throughout the whole experiment.

CtxtFC was conducted in two trials: conditioning and test trials, performed 24 h apart. On the conditioning day, MSDB sham or lesioned animals were trained so as to associate footshocks to the context, as published in literature^56^. The conditioning sessions involved the following steps: (a) exploration of the conditioning chamber for 110 s (b) administration of three footshocks (2 s, 1.5 mA) that were delivered at regular interval (110 s; *3 shock* group). After the final shock, the animal was left in the experimental chamber for another 30s before being removed. As a control experiment, some animals were allowed to explore the conditioning chamber for 366 s without receiving any footshocks (*No shock* condition). On test day, each animal was re-exposed to the context for 480 s. The extent of freezing was analysed by the software and expressed as a percentage. Freezing threshold was set at 3 s i.e. the animal had to cease movement for at least 3 s for an episode to be registered as freezing. In addition to freezing, the animal’s ambulation, especially those which occurred when footshocks were administered, were recorded during fear conditioning training. Thus, animal’s ambulation and the corresponding velocity were taken as its response to footshock.

### Cellular investigations

#### Formalin-induced expression of pERK

Separate experiments were performed to examine the effect of KA-induced neural destruction on the formalin (2.5%)-induced expression of pERK in ACC. The animals were sacrificed 15 min after formalin injection into hind paw. This time point corresponds to the period of robust expression of pERK following formalin injection^18^. In experiments which investigated the effects of systemic morphine on formalin-induced induction of pERK in the ACC, the animals were habituated as described above. On the day of formalin test, morphine (5 mg/kg, Sigma) or vehicle was administered via i.p followed immediately by right hind paw injection of 2.5% formalin. The animal was left in the formalin observation chamber and was sacrificed 15 min after hind paw injection. No behavioural observations were made for these groups of animals.

#### Retrieval-induced expression of Egr-1/Zif268

All CPA and ctxtFC animals were sacrificed 2 h after memory retrieval on test day so as to investigate the extent of Egr-1/Zif268 induced in the amygdala and hippocampus. For CPA experiments, additional controls include:

- Home cage controls to determine the constitutive levels of Egr-1/Zif268 (*Basal*);
- Formalin (*2.5%F*) injected animals (sacrificed 2 h after injection) to determine the formalin-induced expression of Egr-1/Zif268, if any; and
- Non-retrieval animals (*Non-ret*) to determine the effect of the conditioning procedure *per se*. Thus, these animals were trained with the F-CPA protocol but sacrificed in the home cage without F-CPA retrieval.

### Immunohistochemistry and histology

The immunohistochemical-labelling of brain tissue was performed as described previously^9^,^57^,^58^. Briefly, after perfusion and fixation with 4% paraformaldehyde (Sigma), the brain was sectioned into 60-μm coronal sections using a vibratome (Leica VT1200, Leica Microsystems GmbH, Wetzlar, Germany). Alternate sections were collected from the regions of interest and immunolabelled with the respective antibodies (see Table 1). Briefly, (a) the rACC region was labelled for pERK and (b) the amygdaloid and hippocampal regions were labelled for Egr-1/Zif268. In addition, so as to estimate the extent of lesion, (a) the amygdaloid region was labelled for CD11b. CD11b immunohistochemistry was used to demarcate the gliotic area arising due to ibotenic acid-induced damage in amygdala, and (b) the medial septal region was labelled for both choline acetyltransferase (ChAT) and parvalbumin (PV) to quantify the extent of KA-induced destruction of each neuronal population in the region. The avidin-biotin complex (ABC, Vector Laboratories Inc., Burlingame, CA) method was used for antigen detection.

In drug microinjection experiments, animals were perfused and the brains fixed in 10% formalin (Merck). Coronal sections of 60–100 μm thickness were taken through the septal region of the fixed brain and were stained with 0.5% cresyl violet (Sigma) for identification of microinjection site.

### Data analyses

#### Electrophysiological data

Electrophysiological data were digitized (field activity at 256 Hz) and collected using Spike2 software (CED) for offline analyses. The following parameters were analysed: (a) time course of change in duration of theta wave activity (s/5 min) and (b) time course of change in FFT peak power in theta range (4–12 Hz).

During analysis, the EEG trace was digitally band pass filtered at 1–40 Hz (FIR filter). Data were calculated using artefact-free field activity traces. Duration of theta was calculated by computing the period of time (seconds per 5-min block) for which theta was visually identified as a continuous sinusoidal oscillation of at least 1s duration at frequencies of 4–12 Hz. FFT analyses (frequency resolution of 0.5 Hz) was also performed on theta wave activity (minimum of 2s of visually identified theta wave activity) recorded during the 60-min of open field experiment. The FFT was computed in blocks of 5 min. As described previously^9^, the FFT theta power, presented as units of mV^2^ by the program was normalised by dividing with 0.086 to give the power (peak-to-peak amplitude square in the mV^2^ unit) of the field activity. For statistical analyses, the theta peak power recorded during open field was normalised against the peak theta power recorded during exploration just before the commencement of the open field experiment.

#### Quantification of behaviour

In the formalin test, behaviours were quantified in 5-min blocks over the 90-min observation period. The following behaviours were quantified: (a) the duration of licking, (b) the number of flinches of the injected paw and (c) the ambulatory distance.

#### Quantification of immunolabelled neurons

The method of quantification of immunolabelled neurons was as described previously^9^,^59^. Briefly, the histological sections were digitised at 750 d.p.i. using an imaging software (Montage Explorer) under 4X magnification (Nikon Eclipse E408 light microscope). Subsequently, immunolabelled neurons, including Egr-1/Zif268-, pERK-, ChAT- and PV-ir neurons, were counted with an analysis software (MCID^TM^ Analysis, 7.1, UK). The following criteria were adopted for identification of labelled cells^9^,^59^: (a) the intensity of immunolabelled neurons was at least 300% more intense than the average intensity through the region, (b) the area of the label was >10 pixels for cytoplasmic label (pERK, Egr-1/Zif268, ChAT and PV), and > five pixels for nuclear label (Egr-1/Zif268; 1 pixel:5 μm^2^) and (c) form factor was >0.8 for nuclear label (form factor is a measure of degree of roundness, 1 indicates a perfectly round target).

The rACC (~AP 4.20–2.50 mm) was counted for number of pERK-ir neurons. The basolateral amygdala (BLA; ~AP −2.20–−3.60 mm) and dorsal hippocampus field CA1 (dCA1; ~AP −2.20–−4.50 mm) were counted for Egr-1/Zif268-ir nuclei. The medial septal region was divided into medial septum/vertical diagonal band of Broca (MS/VDB) and horizontal diagonal band of Broca (HDB), and counted for the number of ChAT- and PV-ir cells. The anterior-posterior coordinates of the septal sections ranged from ~1.20–0.00 mm anterior to Bregma.

To minimise sampling bias, alternate sections taken through the anterior-posterior extent of a given region were used to count the labelled cells. The total count was averaged for all sections for each region of each animal and then for the experimental group. Percentage destruction of cells was expressed as the number of neurons per section in each KA-treated animal over the average number of neurons per section in the Sham group.

The range of number of sections per animal that were analysed for an immunolabel in a given region are as follows: 17–20 for counting pERK from rACC (average number of sections per animal being 18), 20–22 for counting Egr-1/Zif268 from dorsal hippocampus CA1 (average number of sections per animal being 20), and 12–15 for counting Egr-1/Zif268 from BLA (average number of sections per animal being 12). As regards to the MSDB region, while PV-ir cells were counted for all animals treated with KA, the ChAT-ir neurons were analysed only in the initial part of the study. In context of the latter, alternate sections through the MSDB were stained for PV and ChAT, respectively. The number of sections analysed for each marker per animal ranged from 10–12. On average 11 sections were analysed for each marker per animal.

### Statistical analyses

Results are expressed as mean ± SEM. The ‘n’ number for each experiment is the number of animals used for that experiment. The data were analysed using one of the following statistical tests (Prism 5, GraphPad Software Inc.): (a) two-way repeated measures (RM) ANOVA. This test was used to analyse the time course of effect of a treatment, (b) one-way ANOVA. This test was used to compare multiple groups, and (c) two-tailed unpaired t-test, which was used to compare two groups. In some instances, where Bartlett’s test showed unequal variance, the data were normalised by log transformation and then analysed. Post-hoc analyses after two-way RM ANOVA and one-way ANOVA were carried out using Bonferroni and the Newman Keuls tests for multiple comparisons, respectively. Statistical significance was accepted at p ≤ 0.05.

## Additional Information

**How to cite this article**: Ang, S. T. *et al.* GABAergic neurons of the medial septum play a nodal role in facilitation of nociception-induced affect. *Sci. Rep.* **5**, 15419; doi: 10.1038/srep15419 (2015).

## Figures and Tables

**Table 1:** List of antibodies for immunohistochemistry.

**Brain region**	**Primary antibody**	**AP coordinates of the sections**	**Source of antibody**	**Antibody dilution**	**Secondary antibody (biotinylated)**	**Source of antibody**	**Antibody dilution**
To quantify extent of lesion:							
Medial septum	**Mouse monoclonal anti-parvalbumin (PV)** Detects ~12 kDa parvalbumin protein	~1.20–0.00	Sigma, USA P3088 Lot #035M4879V	1:2000	Goat anti-mouse IgG	Calbiochem, USA	1:400
	**Rabbit polyclonal anti-choline acetyltransferase (ChAT)** Detects ~70 kDa choline acetyltranferase protein in cholinergic neurons	~1.20–0.00	Cedarlane, Canada CLN118 Lot # LH812Y	1:1600	Goat anti-rabbit IgG	Calbiochem, USA or Sigma, USA	1:1000
Amygdala	**Mouse polyclonal anti-rat CD11b** Detects the receptor for the iC3b component of complement in macrophages, dendritic cells, granulocytes and microglial cells in brain	~−2.20–−3.60	AbD Serotec, UK MCA275G	1:1000	Horse anti-mouse IgG	Vector Laboratories, USA	1:1000
To investigate effects of MS lesion on cellular responses:							
Rostral anterior cingulate cortex	**Rabbit monoclonal anti-phospho-p44/42 MAPK (Erk1/2)** Detects dually phosphorylated Erk1/2 at Thr 202 & Tyr204/Thr185 & Tyr187	~5.20–2.20	Cell Signaling, USA #4370 Ref # 05/2014	1:1500	Goat anti-rabbit IgG	Calbiochem, USA or Sigma, USA	1:1000
Amygdala Hippocampus	**Rabbit polyclonal anti-early growth response 1 (Egr1/Zif268)** Detects ~82 kDa Erg-1 epitopes on C-terminus	~−2.20–−3.60 ~−2.00–−4.50	Santa Cruz, USA sc-110 Lot # K1714	1:1000			
